# Genome Structural Diversity among 31 *Bordetella pertussis* Isolates from Two Recent U.S. Whooping Cough Statewide Epidemics

**DOI:** 10.1128/mSphere.00036-16

**Published:** 2016-05-11

**Authors:** Katherine E. Bowden, Michael R. Weigand, Yanhui Peng, Pamela K. Cassiday, Scott Sammons, Kristen Knipe, Lori A. Rowe, Vladimir Loparev, Mili Sheth, Keeley Weening, M. Lucia Tondella, Margaret M. Williams

**Affiliations:** a Division of Bacterial Diseases, Centers for Disease Control and Prevention, Atlanta, Georgia, USA; b Biotechnology Core Facility Branch, Division of Scientific Resources, Centers for Disease Control and Prevention, Atlanta, Georgia, USA; c Vermont Department of Health Laboratory, Burlington, Vermont, USA; Swiss Federal Institute of Technology Lausanne

**Keywords:** *Bordetella pertussis*, genome rearrangements, optical mapping, pertactin, whole-genome sequencing

## Abstract

During 2010 and 2012, California and Vermont, respectively, experienced statewide epidemics of pertussis with differences seen in the demographic affected, case clinical presentation, and molecular epidemiology of the circulating strains. To overcome limitations of the current molecular typing methods for pertussis, we utilized whole-genome sequencing to gain a broader understanding of how current circulating strains are causing large epidemics. Through the use of combined next-generation sequencing technologies, this study compared *de novo*, single-contig genome assemblies from 31 out of 33 *Bordetella pertussis* isolates collected during two separate pertussis statewide epidemics and 2 resequenced vaccine strains. Final genome architecture assemblies were verified with whole-genome optical mapping. Sixteen distinct genome rearrangement profiles were observed in epidemic isolate genomes, all of which were distinct from the genome structures of the two resequenced vaccine strains. These rearrangements appear to be mediated by repetitive sequence elements, such as high-copy-number mobile genetic elements and rRNA operons. Additionally, novel and previously identified single nucleotide polymorphisms were detected in 10 virulence-related genes in the epidemic isolates. Whole-genome variation analysis identified state-specific variants, and coding regions bearing nonsynonymous mutations were classified into functional annotated orthologous groups. Comprehensive studies on whole genomes are needed to understand the resurgence of pertussis and develop novel tools to better characterize the molecular epidemiology of evolving *B. pertussis* populations.

**IMPORTANCE** Pertussis, or whooping cough, is the most poorly controlled vaccine-preventable bacterial disease in the United States, which has experienced a resurgence for more than a decade. Once viewed as a monomorphic pathogen, *B. pertussis* strains circulating during epidemics exhibit diversity visible on a genome structural level, previously undetectable by traditional sequence analysis using short-read technologies. For the first time, we combine short- and long-read sequencing platforms with restriction optical mapping for single-contig, *de novo* assembly of 31 isolates to investigate two geographically and temporally independent U.S. pertussis epidemics. These complete genomes reshape our understanding of *B. pertussis* evolution and strengthen molecular epidemiology toward one day understanding the resurgence of pertussis.

## Abstract (precis)

Pertussis, or whooping cough, is the most poorly controlled vaccine-preventable bacterial disease in the United States, which has experienced a resurgence for more than a decade. Once viewed as a monomorphic pathogen, *B. pertussis* strains circulating during epidemics exhibit diversity visible on a genome structural level, previously undetectable by traditional sequence analysis using short-read technologies. For the first time, we combine short- and long-read sequencing platforms with restriction optical mapping for single-contig, *de novo* assembly of 31 isolates to investigate two geographically and temporally independent U.S. pertussis epidemics. These complete genomes reshape our understanding of *B. pertussis* evolution and strengthen molecular epidemiology toward one day understanding the resurgence of pertussis.

## INTRODUCTION

*Bordetella pertussis* is the causative agent of whooping cough (pertussis), a respiratory disease affecting all age groups, but with the highest disease severity in unvaccinated infants. Whole-cell vaccines against pertussis were introduced in the United States during the 1940s, and greatly reduced disease incidence, but were replaced during the 1990s by acellular vaccines, which produced less-severe side effects (1–4). In addition to diphtheria and tetanus toxoids, the entire childhood vaccination series of diphtheria–tetanus–acellular-pertussis (DTaP) vaccine contains inactivated pertussis toxin (Ptx) and one or more additional virulence-related bacterial components: filamentous hemagglutinin (Fha), pertactin (Prn), or fimbria (Fim) types 2 and 3. Additionally, a single dose of the adolescent and adult booster Tdap (diphtheria, tetanus, and acellular-pertussis vaccine) was recommended in 2005 to counteract the rise of reported cases in adolescents and adults (3, 5, 6). Despite availability, administration, and high coverage of the acellular vaccine reported in the country as a whole, pertussis cases in the United States over the past decade have increased to record numbers not seen since the 1950s. Many states have experienced epidemic levels of pertussis cases in recent years, specifically California in 2010 and Washington and Vermont in 2012 (7–11). In 2010, California reported over 9,000 cases (23.4 cases per 100,000 residents), while in 2012 Vermont reported over 600 cases (103 cases per 100,000 residents), over 10 times more than average for that time of year (7, 10). With incidence being highest in unvaccinated infants younger than 6 months and fully vaccinated preadolescents (7 to 10 years) in California, previous studies concluded that unprotected infants were at the highest risk for disease, while waning protection from the childhood acellular vaccine series contributed to disease in preadolescents (7, 12). While there is no single explanation for this resurgence of cases, it has been ascribed to many factors such as pathogen adaptation, improved surveillance and laboratory diagnostics, and waning protection and immune response provided by the acellular vaccine (12–14).

Since 2010, the U.S. population of circulating *B. pertussis* strains has increasingly become deficient in Prn, an acellular vaccine component, due to various mutations, primarily the disruption of *prn* by the mobile genetic element IS*481* (11, 14–18). In 2012, 92% of isolates collected in Vermont did not produce pertactin, suggesting that a selective advantage to *prn* deficiency may have played a role in that epidemic (14). In separate global locations, two isolates lacking Ptx have been obtained recently, one of which is also Prn deficient (19, 20). The loss of vaccine immunogens identified by current molecular typing methods provides strong evidence that current populations of *B. pertussis* no longer reflect the genotypic profile of vaccine strains and could be adapting to the immune response elicited by the acellular vaccine (11, 14, 15, 19, 21–24). This, along with data supporting higher rates of evolution in vaccine antigen genes, has generated concern that other genes encoding vaccine components may harbor mutations not identifiable through current molecular typing techniques (25). Additionally, the recent analysis of a statewide pertussis epidemic in Washington during 2012 revealed that pulsed-field gel electrophoresis (PFGE), a whole-genome restriction digest analysis, was the most powerful indicator of diversity in this population of circulating pertussis strains (11). With evidence suggesting that current strains exhibit genomic diversity that nucleotide variation identified by short-read sequencing fails to explain, it is of great interest to employ long-read technologies to detect mutations and assess the role of genome structure in pertussis resurgence (11, 15, 26).

With hundreds of insertion sequences (ISs) and repeat regions in current circulating strains of *B. pertussis*, whole-genome sequencing is challenging. There are currently over 400 *B. pertussis* draft genomes available in public databases, all of which include at least 200 contigs assembled from short-read sequencing. Such fragmented assemblies provide limited information about genome arrangement or gene order and are, therefore, not suitable for evaluating contributions of rearrangement diversity toward pertussis incidence, resurgence, or strain evolution. Complete, *de novo* genome assembly of *B. pertussis* is essential in pursuit of this information and now possible with long-read (>1-kb) sequencing and whole-genome mapping (27, 28). The recent release of complete genomes highlights the contrast between modern pertussis strains and current vaccine references, at both the nucleotide and the structural levels (29, 30). In an effort to better understand pertussis resurgence and evolution, here we characterize genome structure and provide direct evidence of large genome rearrangements in annotated, single-contig genome assemblies from 31 isolates collected during two geographically and temporally distinct pertussis epidemics. This depth of resolution facilitates analysis that is not possible through conventional molecular typing or short-read sequencing. The availability of these complete genomes will hopefully aid future transcriptomics and proteomics pursuits toward a deeper understanding of pertussis reemergence and increased incidence.

## RESULTS

### Reference-free assembly of complete genomes.

Through the use of Pacific Biosciences (PacBio) RSII and Illumina MiSeq sequencing technologies, 33 genomes (31 epidemic, 2 vaccine) (Table 1) were *de novo* assembled into single contigs. All genomes were approximately 4.1 Mb in size with an average G+C content of 67.7%. Detailed assembly metrics for each genome are outlined in Data Set S1 in the supplemental material. The genome assembly of I475 provided no circular overlap to ensure closing of the single contig, while the genome assemblies for I488 and I517 failed one or more validation steps, resulting in multiple contig(s). Annotation information, including the number of genes, coding sequences (CDS), pseudogenes, frameshifted genes, and insertion sequences, is outlined in Data Set S1 in the supplemental material. In addition, all genomes contained 51 tRNAs and 3 rRNA operons. Tohama I (E476) and the Chinese vaccine (CS) strain (C393) resequenced in this study were 16.8 kb and 9.5 kb larger than the NCBI reference genomes Tohama I and CS, respectively, due to the discovery of additional IS*481* copies (see Data Set S1).

10.1128/mSphere.00036-16.2 Data Set S1 Assembly results for 2 vaccine strains and 33 isolates collected during the California (CA) 2010 and Vermont (VT) 2012 statewide pertussis epidemics that were selected for whole-genome sequencing. Download Data Set S1, XLSX file, 0.1 MB. Copyright © 2016 Bowden et al. 2016 Bowden et al. This content is distributed under the terms of the Creative Commons Attribution 4.0 International license.

**TABLE 1:** Metadata for *B. pertussis* isolates collected during the California 2010 and Vermont 2012 statewide epidemics and vaccine strains that were selected for whole-genome sequencing^l^

Isolate	Location	Yr of isolation	Age at symptom onset	Vaccination status	PFGE type	MLVA^a^	*prn*^b^	*ptxP*^b^	*ptxA*^b^	*fimH*^b^,^c^	*prn* genotype	Prn production^d^
H374	CA	2010	Infant	UV	CDC002	16	2	3	1	1	WT *prn*	+
H375^e^	CA	2010	Infant	UV	CDC268	186	1	1	2	1	Del nt 26-109^f^	−
H378	CA	2010	Infant	UV	CDC253	27	2	3	1	1	IS*481*::240 Fwd^g^	−
H379	CA	2010	Infant	UV	CDC046	27	2	3	1	2	WT *prn*	+
H380^e^	CA	2010	Infant	UV	CDC013	27	2	3	1	2	WT *prn*	+
H489	CA	2010	Infant	UV	CDC082	27	2	3	1	2	WT *prn*	+
H542^e^	CA	2010	Infant	UV	CDC269	27	2	3	1	1	WT *prn*	+
H559^e^	CA	2010	Infant	UV	CDC253	27	2	3	1	1	IS*481*::240 Fwd^g^	−
H561	CA	2010	Infant	UV	CDC170	16	2	3	1	2	WT *prn*	+
H563^e^	CA	2010	Infant	≥1 dose	CDC271	27	2	3	1	2	WT *prn*	+
H564	CA	2010	Child	UV	CDC013	27	2	3	1	2	WT *prn*	+
H622	CA	2010	Infant	UV	CDC217	27	2	3	1	1	WT *prn*	+
H627	CA	2010	Infant	UV	CDC217	27	2	3	1	1	WT *prn*	+
H788	VT	2011	Infant	UV	CDC046	128	2	3	1	2	WT *prn*	+
I669	VT	2011	Adult	UV	CDC013	27	2	3	1	2	WT *prn*	+
I468	VT	2012	Child	UV	CDC002	27	2	3	1	1	SC @ nt 1273	−
I469	VT	2012	Child	≥1 dose	CDC342	27	2	3	1	2	IS*481*::1613 Rev^h^	−
I472	VT	2012	Adolescent	UV	CDC046	27	2	3	1	2	IS*481*::1613 Rev^h^	−
I475	VT	2012	Adult	UV	CDC237	27	2	3	1	1	IS*481*::1613 Fwd^i^	−
I476	VT	2012	Adolescent	UTD	CDC300	27	2	3	1	1	IS*481*::1613 Fwd^i^	−
I480	VT	2012	Child	≥1 dose	CDC217	27	2	3	1	1	WT *prn*	+
I483	VT	2012	Infant	UTD	CDC237	27	2	3	1	1	IS*481*::1613 Fwd^i^	−
I488	VT	2012	Child	≥1 dose	CDC343	27	2	3	1	1	IS*481*::1613 Fwd^i^	−
I496	VT	2012	Child	UTD	CDC343	27	2	3	1	1	IS*481*::1613 Fwd^i^	−
I498	VT	2012	Adult	≥1 dose	CDC253	27	2	3	1	1	IS*481*::240 Rev^j^	−
I517	VT	2012	Child	≥1 dose	CDC344	27	2	3	1	1	IS*481*::1613 Fwd^i^	−
I518	VT	2012	Child	UTD	CDC002	27	2	3	1	1	SC @ nt 1273	−
I521	VT	2012	Child	≥1 dose	CDC237	27	2	3	1	1	IS*481*::1613 Fwd^i^	−
I538	VT	2012	Child	UNK	CDC002	27	2	3	1	1	*prnP* (–74 nt)^k^	−
I539	VT	2012	Child	UTD	CDC002	27	2	3	1	1	*prnP* (–74 nt)^k^	−
I646	VT	2012	Child	UV	CDC274	27	2	3	1	1	WT *prn*	+
I656	VT	2012	Child	UTD	CDC010	27	2	3	1	1	WT *prn*	+
I707	VT	2012	Child	UTD	CDC253	27	2	3	1	1	WT *prn*	+
C393	China	1951	UNK	NA	CDC052	UNK	1	1	2	1	WT *prn*	+
E476	Japan	1954	UNK	NA	CDC232	38	1	1	2	1	WT *prn*	+

a MLVA type is defined by the repeat counts for VNTR1, VNTR3, VNTR4, VNTR5, and VNTR6 (21).

b Single-copy locus (21).

c Formerly referred to as *fim3.*

d Based on enzyme-linked immunosorbent assay.

e Fatal cases.

f Signal sequence deletion (nt 26 to 109).

g IS*481* forward insertion at nt 240.

h IS*481* reverse insertion at nt 1613.

i IS*481* forward insertion at nt 1613.

j IS*481* reverse insertion at nt 240.

k Promoter disruption (–74 nt), previously identified as promoter inversion (11, 15).

l Abbreviations: MLVA, multilocus variable-number tandem-repeat analysis; CA, California; VT, Vermont; nt, nucleotide; UTD, up to date; UV, unvaccinated; UNK, unknown; WT, wild-type; SC, stop codon; NA, not applicable.

### Large-scale genome rearrangements.

**FIG 1:**
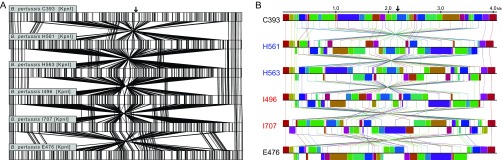
Large-scale genome rearrangements within a subset of California (H561 and H563, blue) and Vermont (I496 and I707, red) epidemic isolates compared to vaccine strains E476 and C393 as visualized by whole-genome restriction mapping and alignment with MapSolver (A) and genome sequence alignment with progressiveMauve (B). Connecting lines indicate either conserved restriction fragments (A) or homologous sequence blocks (B). Sequence maps begin at the replication origin, and the approximate replication terminus is indicated with an arrow.

Multiple insertions, deletions, and inversions were seen in all genomes of epidemic isolates relative to E476 and C393 using both optical mapping and whole-genome sequence alignment (Fig. 1). Such genome structural diversity was also observed within epidemic isolates, which included 16 discrete, large-scale architectural profiles. The 13 California genomes comprised 10 rearrangement profiles, and 11 profiles were seen in the 18 Vermont genomes. Further, only five structural profiles were observed in both states, with five and six additional profiles distinct to California and Vermont, respectively (examples of these structural profiles can be found in Fig. S1B in the supplemental material).

10.1128/mSphere.00036-16.1 Figure S1  Genome structural variation among epidemic isolates from California (blue) and Vermont (red). (A) Maximum-likelihood phylogenetic reconstruction based on the order and orientation of conserved, colinear genome fragments identified by whole-genome alignment. (B) Alignment of 14 select genomes with different structural architectures (highlighted with asterisks in panel A). Connected blocks of the same color represent conserved regions, and flags indicate the presence of an IS*481* (black), IS*1002* (blue), or rRNA operon (red) at the boundary between colinear regions. Download Figure S1, TIF file, 0.4 MB. Copyright © 2016 Bowden et al. 2016 Bowden et al. This content is distributed under the terms of the Creative Commons Attribution 4.0 International license.

**FIG 2:**
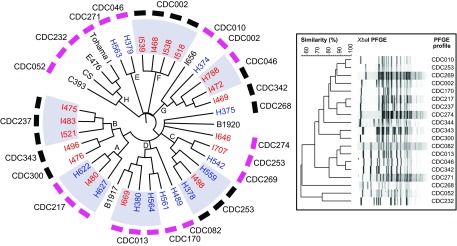
Maximum likelihood for gene order (MLGO) clustering of epidemic strains from the 2010 California (blue) and 2012 Vermont (red) statewide pertussis epidemics. Publicly available genomes (Tohama I, CS, B1917, and B1920) and two resequenced vaccine strains (C393 and E476) were also included (black). Pertactin production and deficiency of each strain are indicated by pink and black boxes, respectively. Clades referenced within the text are indicated by letters. The inset displays banding patterns of the PFGE profiles of all epidemic and vaccine strains sequenced in this study. The dendrogram of PFGE profiles was created using the unweighted pair group method using average linkages (UPGMA) with 1% band tolerance and optimization settings. Genomes for the epidemic isolates I488 and I517 were excluded from the analysis (see Materials and Methods and Data Set S1 in the supplemental material).

To visualize the dynamic relationship between the vaccine strains and isolates collected during the California and Vermont epidemics resulting from genome structural changes, a Maximum Likelihood for Gene Order (MLGO) tree was constructed from a multiple sequence alignment (Fig. 2). This clustering revealed eight major clades (A to H) that distinguished the epidemic isolates and vaccine strains based on gene rearrangement and gene order (Fig. 2). Isolates with the same or similar PFGE profiles clustered by genome rearrangement (Fig. 2). The vaccine strains formed a distinct clade (H) which is distant from the U.S. epidemic isolates and B1917 (CP009751) and B1920 (CP009752) (29). B1917, the *ptxP3* lineage strain corresponding to current circulating strains, clusters with isolates in clade D, while B1920, the *ptxP1* lineage strain predominant from 1960 to the 1990s, is no more closely related to I646 and the only *ptxP1* epidemic isolate (H375) than it is to the rest of the epidemic and vaccine strain genomes (Fig. 2) (29). Four clades (B, D, E, and F) show clustering of epidemic isolates by state and *prn* production status. Further, the two isolates collected in Vermont during 2011 (H788 and I669) are diverse and not closely related to each other by genome structure (Fig. 2).

### Rearrangement boundaries.

To better understand what mediates observed rearrangements, the genome content at each predicted rearrangement boundary was investigated further. When comparing California and Vermont isolates alone, 26 conserved homologous blocks (≥1.5 kb) were identified in these 31 genomes, separated by 25 predicted rearrangement boundaries (Table 2; see also Fig. S1 in the supplemental material). Eighty percent (*n* = 20) of these boundaries were composed of an IS*481* element (Table 2; see also Fig. S1B). Additionally, 12% (*n* = 3) were composed of an *rrn* operon, while two boundaries were composed of either an IS*1002* element or a combination of IS*1002* and IS*481* (Table 2; see also Fig. S1B). When two vaccine strains (C393 and E476) were included in the alignment, all additional predicted rearrangement boundaries contained IS*481* (Table 2). This larger comparison identified 45 conserved homologous blocks (≥1.5 kb), separated by 44 predicted rearrangement boundaries, 89% (*n* = 39) of which included copies of IS*481* (Table 2).

**TABLE 2:** Characteristics of rearrangement boundaries

Characteristic	Value for strain type:	
	Epidemic	Epidemic and vaccine
No. of genomes	31^a^	33^a^
No. of rearrangement boundaries	25	44
No. of copies/genome		
IS*481* (≥231)	20	39
rRNA (3)	3	3
IS*1002* (4)	1	1
IS*1002/*IS*481*	1	1

a I488 and I517 excluded from analysis.

### Virulence gene comparisons.

The sequences of 44 virulence-related genes for all epidemic genomes and C393 were compared to E476 (see Data Set S2 in the supplemental material). The resulting alignments revealed that 30 of these virulence-related genes showed no sequence differences relative to E476. Gene sequences extracted from the assemblies for *prn*, *ptxP*, *ptxA*, and *fimH* (*fim3*) exhibited the same allelic variations previously identified through multilocus sequence typing (MLST) and *prn* sequence typing by PCR (Table 1; see also Data Set S2). Unique single nucleotide polymorphisms (SNPs) identified in virulence-related genes of the epidemic isolates are displayed in Table 3 and in Data Set S2 in the supplemental material. Six SNPs in virulence-related genes were found to be unique to a single isolate, while two were identified in all epidemic isolates, including C393 (Table 3). Most of the SNPs identified (7/10) resulted in an amino acid change (Table 3). Interestingly, a majority of SNPs in virulence-related genes were found in GC-rich regions (Table 3). Additionally, no epidemic genomes harbored the 23S A2047G mutation known to produce erythromycin/azithromycin resistance (31–33).

10.1128/mSphere.00036-16.3 Data Set S2 Sequence comparison of virulence-related genes in *B. pertussis* epidemic isolates compared to E476 (Tohama I). Download Data Set S2, XLSX file, 0.1 MB. Copyright © 2016 Bowden et al. 2016 Bowden et al. This content is distributed under the terms of the Creative Commons Attribution 4.0 International license.

**TABLE 3:** Unique SNPs in virulence-related genes^a^ of *B. pertussis* epidemic isolates compared to E476 (Tohama I)

Gene	Isolate(s)	SNP location	Region characteristic	Amino acid change	Protein expressed? (49)	Reference(s)
*bapC*	H374	655 A→G	String of 2 G’s	219 Asp→Gly	Unknown	This study
*bfrD*	H564	1634 G→A	GC-rich region	545 Ser→Asn	Unknown	This study
*bipA*	H563	1070 G→T	GC-rich region	357 Arg→Leu	Unknown	This study
*brkA*	I646	640 C→T	GC-rich region	214 Pro→Ser	Unknown	This study
*bscC*	H375	1677 G→A	String of 2 A’s	NA^b^	Yes	This study; 49
*bvgR*	C393	36 C→T	String of 3 T’s	NA	Unknown	This study; 23
*bvgS*	All epidemic isolates, C393	2113 A→G	GC-rich region	705 Lys→Glu	Yes	47, 49, 50, 51, 52
*fimD*	All epidemic isolates, C393	356 T→C	GC-rich region	119 Phe→Ser	Yes	47, 49, 50, 53, 54
*ptxB*	All epidemic isolates	133 G→A	GC-rich region	45 Gly→Ser	Yes	47, 49, 50, 55
*ptxC*	All epidemic isolates except H375	681 C→T	String of 2 T’s	NA	Yes	47, 49, 56

a Does not include previously identified SNPs associating with MLST typing loci.

b NA, not applicable.

### Variant analysis.

**FIG 3:**
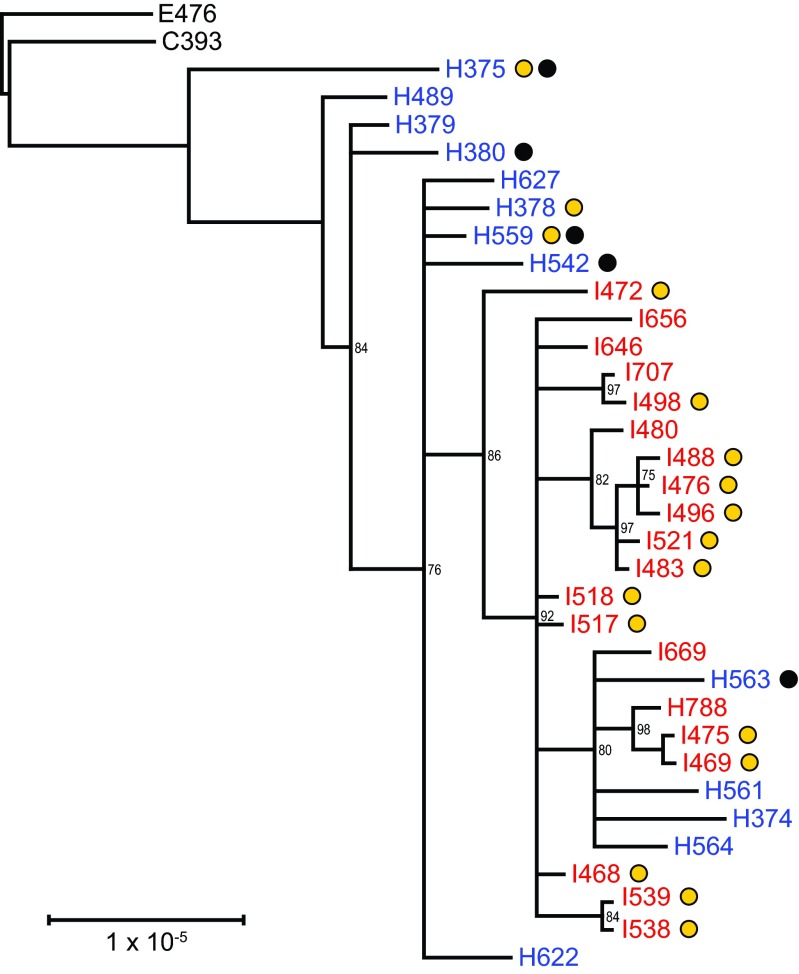
Maximum likelihood phylogenetic reconstruction from the concatenated alignment of 408 variable sites (SNVs and MNVs). Vaccine strains resequenced in this study are indicated in black, California isolates are indicated in blue, and Vermont isolates are indicated in red. Yellow circles denote pertactin-deficient strains, and black circles denote strains recovered from fatal cases. The scale bar indicates number of substitutions per site and has been corrected for ascertainment bias based on the nucleotide composition of invariant sites in E476. All epidemic genomes were included in this analysis.

The phylogenetic relationships among epidemic isolates were reconstructed from a concatenated alignment of 408 variable sites (Fig. 3). There was greater diversity among the 13 California isolates (Fig. 3), with H375 being most divergent, and no clustering was observed among isolates recovered from fatal cases (Fig. 3). In contrast, Vermont isolates exhibited more homogeneity (Fig. 3). Additionally, four California genomes (H563, H561, H374, and H564) clustered among the earliest collected Vermont genomes, three of which (H788, I475, and I669) were collected from unvaccinated patients (Fig. 3). Pertactin-deficient and pertactin-producing isolates are dispersed throughout the tree (Fig. 3). The two isolates with the *prn* promoter disruption (I538 and I539) are exclusive to one clade (Fig. 3; Table 1).

**FIG 4:**
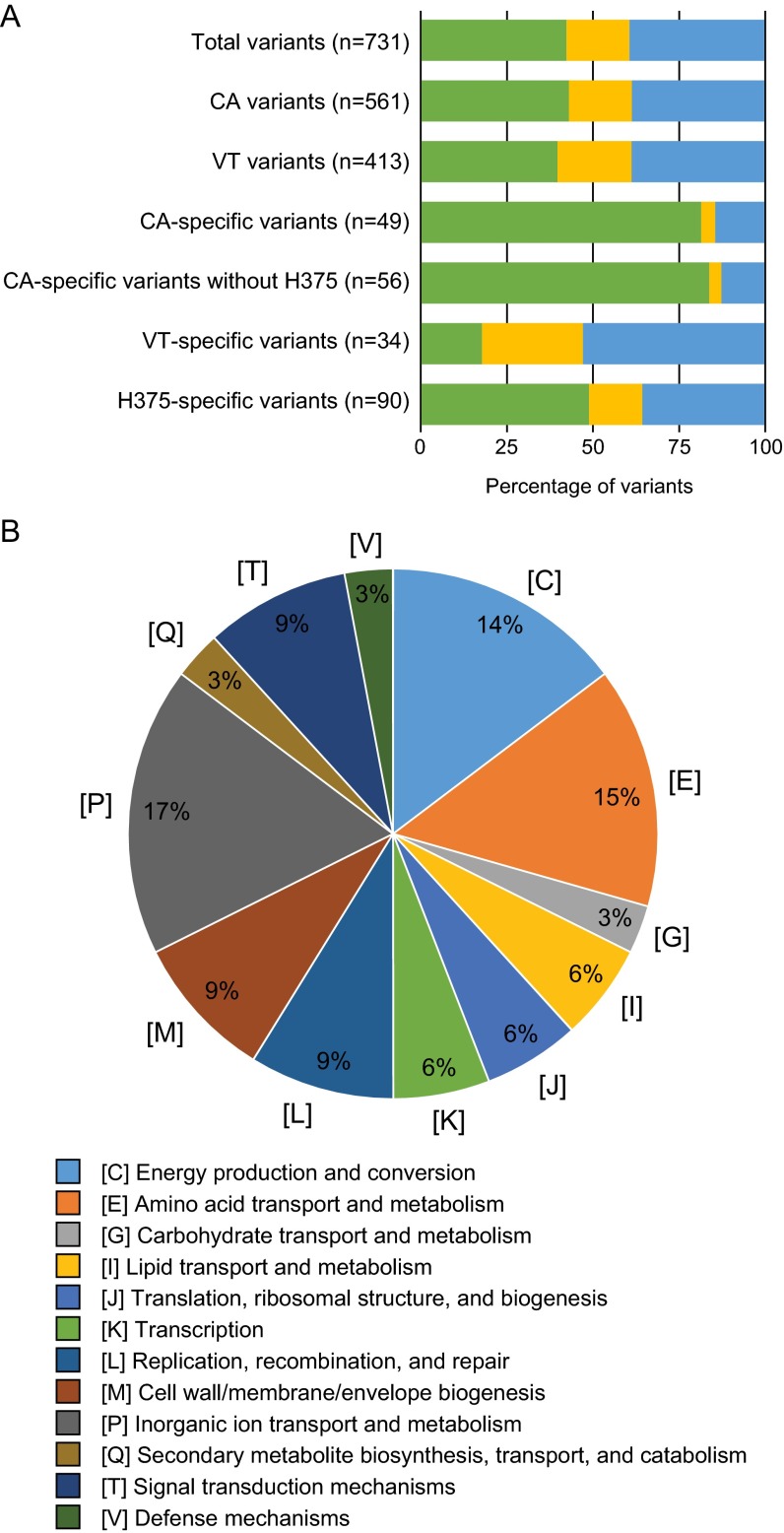
Sequence variants within epidemic genomes. (A) Distribution of noncoding (green), synonymous (yellow), and nonsynonymous (blue) variants. (B) Functional classification of predicted protein sequences with observed nonsynonymous mutation in at least 26 epidemic genomes categorized according to EggNOG v.4. Variants were statistically significant by Fisher's exact test.

To characterize putative phenotypic effects of observed nucleotide variants, all mutations were classified as noncoding, synonymous, or nonsynonymous, as seen in Fig. 4. Within the 33 epidemic genomes analyzed, a total of 731 variants were discovered and 39.4% (*n* = 288) were identified as nonsynonymous (Fig. 4A; see also Data Set S3 in the supplemental material). Of these, 34 unique protein-encoding genes were mutated in at least 26 of the 33 epidemic genomes and 17% were found to be involved in inorganic ion transport and metabolism (Fig. 4B; see also Data Set S4). Epidemic genomes were divided by state and characterized in the same manner to identify state-specific mutations. California epidemic genomes contained more variants than Vermont (561 and 413, respectively), with a majority of noncoding mutations (82%) specific to California genomes and a majority of nonsynonymous mutations (53%) specific to Vermont epidemic genomes (Fig. 4A; see also Data Sets S5, S6, S7, and S8). The California isolate H375, the only isolate from the *ptxP1* lineage, exhibited the largest number of isolate-specific variants of all epidemic genomes (*n* = 90) and accounted for seven additional noncoding variants upon exclusion from the California-specific variant analysis (Fig. 4A; see also Data Set S3). The genes affected by nonsynonymous mutations specific to each state were further identified (Table 4). In at least nine of the 12 California epidemic genomes, 63% (*n* = 5) of the nonsynonymous mutations were seen in five IS*1663* elements (Table 4; see also Data Set S9). Nine of the 18 nonsynonymous mutations specific to Vermont affected proteins involved in various metabolic pathways (Table 4; see also Data Set S9). Additionally, no variants were found to be exclusive to *prn*-deficient isolates outside the *prn* gene or isolates collected from fatal cases.

10.1128/mSphere.00036-16.4 Data Set S3 Seven hundred thirty-one variants in 33 *B. pertussis* epidemic genomes. Download Data Set S3, XLSX file, 0.1 MB. Copyright © 2016 Bowden et al. 2016 Bowden et al. This content is distributed under the terms of the Creative Commons Attribution 4.0 International license.

10.1128/mSphere.00036-16.5 Data Set S4 Thirty-four nonsynonymous variants in at least 26 of the 33 *B. pertussis* epidemic genomes. Download Data Set S4, XLSX file, 0.1 MB. Copyright © 2016 Bowden et al. 2016 Bowden et al. This content is distributed under the terms of the Creative Commons Attribution 4.0 International license.

10.1128/mSphere.00036-16.6 Data Set S5 Five hundred sixty-one variants in California *B. pertussis* epidemic genomes. Download Data Set S5, XLSX file, 0.1 MB. Copyright © 2016 Bowden et al. 2016 Bowden et al. This content is distributed under the terms of the Creative Commons Attribution 4.0 International license.

10.1128/mSphere.00036-16.7 Data Set S6 Four hundred thirteen variants in Vermont *B. pertussis* epidemic genomes. Download Data Set S6, XLSX file, 0.1 MB. Copyright © 2016 Bowden et al. 2016 Bowden et al. This content is distributed under the terms of the Creative Commons Attribution 4.0 International license.

10.1128/mSphere.00036-16.8 Data Set S7 Forty-nine California state-specific *B. pertussis* epidemic genome variants. Download Data Set S7, XLSX file, 0.1 MB. Copyright © 2016 Bowden et al. 2016 Bowden et al. This content is distributed under the terms of the Creative Commons Attribution 4.0 International license.

10.1128/mSphere.00036-16.9 Data Set S8 Thirty-four Vermont state-specific *B. pertussis* epidemic genome variants. Download Data Set S8, XLSX file, 0.1 MB. Copyright © 2016 Bowden et al. 2016 Bowden et al. This content is distributed under the terms of the Creative Commons Attribution 4.0 International license.

10.1128/mSphere.00036-16.10 Data Set S9 State-specific nonsynonymous *B. pertussis* epidemic genome variants. Download Data Set S9, XLSX file, 0.1 MB. Copyright © 2016 Bowden et al. 2016 Bowden et al. This content is distributed under the terms of the Creative Commons Attribution 4.0 International license.

**TABLE 4:** State-specific nonsynonymous variants in California and Vermont epidemic *B. pertussis* genomes^a^

Nonsynonymous variant in state	Functional category	Location in E476 (Tohama I)	Variant within gene	Amino acid change	No. of genomes
California					
Transposase for IS*1663*	Replication, recombination, and repair	116940	947 T→A	316 Leu→Gln	13
Transposase for IS*1663*	Replication, recombination, and repair	527395	805 T→C	269 Tyr→His	12
Transposase for IS*1663*	Replication, recombination, and repair	1080959	947 T→A	316 Leu→Gln	9
Transposase for IS*1663*	Replication, recombination, and repair	1090325	947 T→A	316 Leu→Gln	10
Transposase for IS*1663*	Replication, recombination, and repair	1447860	947 T→A	316 Leu→Gln	12
Transposase	Replication, recombination, and repair	3457234	947 A→T	316 Gln→Leu	12
Regulator (hypothetical protein)	Signal transduction mechanisms	3614341	11 G→A	4 Gly→Glu	12
Vermont					
Zinc transporter ZupT	Inorganic ion transport and metabolism	198405	194 T→C	65 Val→Ala	20
FAD/FMN-containing dehydrogenase	Energy production and conversion	216761	19 T→C	7 Ser→Pro	20
RNase E	Translation, ribosomal structure, and biogenesis	489152	2480 C→T	827 Pro→Leu	20
Endoribonuclease l-PSP (RutC family protein)	Function unknown	528567	186 C→G	62 Asp→Glu	20
Diacylglycerol kinase	Lipid transport and metabolism	868389	97 G→A	33 Asp→Asn	19
Glutamate-ammonia-ligase adenylyltransferase	Posttranslational modification, protein turnover, chaperones	1333208	2519 G→A	840 Gly→Asp	20
Aldo-ketoreductase	Function unknown	1388597	625 G→C	209 Ala→Pro	19
Multidrug efflux pump subunit AcrB	Inorganic ion transport and metabolism	2208591	3221 A→G	1074 Asn→Ser	19
Acyltransferase	Function unknown	2271460	24 G→C	8 Gln→His	17
Hypothetical protein	Function unknown	2276856	158 T→C	53 Val→Ala	20
Transposase	Replication, recombination, and repair	2405180	899 C→G	300 Thr→Ser	19
Potassium-transporting ATPase subunit C	Inorganic ion transport and metabolism	2640489	245 T→C	82 Ile→Thr	19
5-Hydroxyvalerate dehydrogenase (HVD)	Energy production and conversion	3011513	1016 C→T	339 Ala→Val	20
NADPH dehydrogenase	Energy production and conversion	3072916	55 A→G	19 Lys→Glu	20
ADP-dependent (S)-NAD(P)H- hydrate dehydratase	Carbohydrate transport and metabolism	3538308	166 G→A	56 Gly→Arg	11
Putative oligopeptide transporter	Function unknown	1189451-1189452	1765_GC_1766	589 Gly fs	19
ATP-cobalamin adenosyltransferase	Coenzyme transport and metabolism	3304784-3304785	545_CCGG_546	183 Ala fs	17
Hypothetical protein	Function unknown	3490857-3490858	1130_CTA_1131	377 Glu delins Asp*	20

a Abbreviations: fs, frameshift; delins, deletion/insertion; FAD, flavin adenine dinucleotide; FMN, flavin mononucleotide; PSP, perchloric acid-soluble protein; *, produces premature stop codon.

To address the issue of vaccine-driven evolution and to determine if certain genes are under selective pressure, the frequencies of nonsynonymous variants and variants within virulence genes across all epidemic genomes were determined. Virulence gene variants had a rate of 0.22 bp/kb compared to 0.24 bp/kb for total epidemic genome variants, with no significant difference between the two. However, nonsynonymous variants had a higher frequency (0.10 bp/kb) than synonymous variants (0.03 bp/kb) (*P* value = 0.0029).

## DISCUSSION

This study illustrates the first effort to evaluate relationships among isolates within and between statewide pertussis epidemics through comparison of complete genome sequences. Prior to the availability of single-contig genomes, PFGE has been the only whole-genome indicator used to fully assess molecular epidemiology and patient transmission of circulating strains of *B. pertussis* (34). To date, analyses of gene order and genome structure heterogeneity have been limited by the resolution of available methods (35, 36). Additionally, few genomes are available with a complete genome assembly that has been confirmed with a second method such as optical mapping. During the process of complete genome assembly, genome optical maps not only provided structural confirmation of assemblies but proved to be useful as a possible genome typing tool with greater detail than that found with PFGE (with approximate location of restriction cut sites mapped). Depending on future questions, it may be beneficial to develop a hierarchical approach, in which optical mapping is first used to compare genome structures as a typing method, followed by genome sequencing if more detail is required.

Here, we generated high-quality genome assemblies and showed their capacity for detecting discrete genome structural variations among two geographically and temporally independent pertussis epidemics at a resolution never seen before. Furthermore, clustering these genomes according to rearrangement patterns revealed correlations with PFGE profiles and associating *prn* mutations. However, no strong association was observed between global genome structure and geography, patient fatality, vaccination status, or time of collection (Fig. 2; Table 1). The genome structures of two vaccine strains resequenced as part of this study differed greatly from epidemic isolates, supporting a growing body of evidence that suggests that current circulating strains of *B. pertussis* have dramatically changed since the isolation of vaccine references (Fig. 2).

Recently, Bart et al. (29) sequenced complete genomes of representative isolates from two predominate global lineages of *ptxP3* and *ptxP1* (B1917 and B1920, respectively) which exhibited structural architectures similar to those of the U.S. epidemic isolates reported here with comparable molecular typing profiles (Table 1; Fig. 2). The finding that B1920 clustered near the only *ptxP1* epidemic isolate may indicate a shift in rearrangement profiles that corresponds to the shift from *ptxP1* and *ptxP3* lineages. Additionally, B1917 and B1920 strains were isolated in 2000 in the Netherlands, temporally and geographically distant from the epidemic isolates sequenced here, suggesting that rearrangement profiles may be stable over time. Although rearrangements have been identified by others, attempts to measure rearrangement rates in *B. pertussis* have been limited by the low resolution of PFGE, and reports of gene order changes following multiple laboratory passages, which differ from natural infection cycles, are conflicting (36–40). Therefore, the historical pattern of genome rearrangement remains unclear. Only through methods like optical mapping and complete genome sequencing, which have been applied to *B. pertussis* U.S. epidemic isolates here for the first time, can these questions be addressed.

Although a diverse number of PFGE profiles among isolates are observed in this and previous studies, the genomic variation underlying this diversity can now be interrogated (11). To do so, we identified genes located at predicted rearrangement boundaries in all fully assembled genomes in this study. The highly conserved *rrn* operons and repetitive mobile elements IS*481* and IS*1002* were found at predicted rearrangement breakpoints in both epidemic and vaccine isolate genomes, the majority of which were composed of IS*481* elements (Table 2; see also Fig. S1 in the supplemental material). Almost all predicted rearrangements were in the form of inversions flanked by inverted sequence repeats, often “symmetric” around the origin or terminus of replication, maintaining replichore balance important for genome stability (41, 42). These data support previous evidence that rearrangements in the *B. pertussis* genome are mediated by insertion sequence (IS) elements through homologous recombination between inverted repeats, presumably during replication, a mechanism long thought to maintain genome plasticity (22, 35, 36, 41, 43, 44).

Aside from rearrangements, mobile elements have been shown to play a role in the creation of pseudogenes, genome reduction, and adaptive evolution toward host specificity (22, 36, 43, 45, 46). Accurate identification of all IS*481*, IS*1002*, and IS*1663* insertions is necessary to evaluate their contribution to genome evolution, and previous studies using short-read sequencing have been unsuccessful (24, 47). Our pipeline provided more robust sequencing data for proper placement of insertion sequences to construct more accurate assemblies, evident when comparing the corrected number of IS*481* elements discovered in the resequenced vaccine strains E476 and C393 and assembly structure errors found when comparing the NCBI CS reference genome with the resequenced C393 (see Data Set S1 in the supplemental material). Alignment of complete genome sequences provides further evidence that IS*481* transposition remains active, consistent with previous reports, but also suggests that this expansion of repetitive sequence may provide additional opportunities for rearrangement (48). With the exception of H561, most Vermont and California epidemic genomes contained more IS*481* copies than both Tohama I and E476. Additionally, further acquisition of insertion sequence copies in currently circulating strains of *B. pertussis* may indicate intraspecies host adaptation through gene inactivation that allows *B. pertussis* to evade protective immunity elicited by the acellular vaccine and cause disease in a vaccinated population. This claim is further supported by studies indicating that the majority of *prn*-deficient U.S. isolates harbor an IS*481* within the *prn* gene locus (11, 15). However, the results of this study suggest that the role of mobile elements in *B. pertussis* genome evolution may not be limited to reduction, raising questions about the potential fitness implications of rearrangement, as others have also speculated (40).

In an effort to identify potential correlates of protection not included in the U.S. acellular vaccine, nonsynonymous mutations were investigated in 40 additional virulence-related genes and throughout the genomes of all epidemic isolates. Many virulence-related gene mutations were identified in isolates collected from infants and children with various effects on amino acid sequence within either a single isolate or all epidemic isolates (Tables 1 and 3) (23, 47, 49–56). Although statistically insignificant due to the small sample size of this study, rates of SNPs in virulence genes seem to support the idea of genetic drift and are not found to occur more frequently than in other genes. However, the rate of nonsynonymous variants suggests that genetic elements might be under selective pressure. The majority of nonsynonymous mutations (53%) were found to affect proteins either identified as transporters or associated with the transport and metabolism of various cellular molecules (Fig. 4B; see also Data Set S4 in the supplemental material). This overrepresentation of SNPs in transport proteins was also seen in a study of SNP density in the global population of pertussis isolates and may provide insight into the mechanism by which current strains have adapted to the current global vaccination state (24). Although state-specific mutations were discovered in IS elements and metabolic proteins in California and Vermont, respectively, it is difficult to make phenotypic implications based on state specificity, disease presentation, or demographic due to the highly biased selection of the small number of isolates chosen for whole-genome sequencing and the lack of a functional assessment in the study. Transcriptomics, protein expression, and functional assays of proteins affected by nonsynonymous mutations in these genomes are needed to fully understand how these mutations affect the ability of *B. pertussis* to infect and cause disease, with varying severity, in the host.

It is clear that we do not fully understand the correlates of protection against *B. pertussis*. However, this study has expanded our understanding of *prn* deficiency, specifically with genomic data visualizing the true nature of the previously predicted promoter inversion and providing evidence that no conserved single or multiple mutational events may be compensating for *prn* deficiency in this collection of isolates, calling into question the role that pertactin plays in pertussis disease (11, 15). This analysis in conjunction with proteomics investigation of several of these isolates, which we are actively pursuing, provides a starting point for determining what proteins are required for disease in certain host populations while identifying potential candidates for future vaccine components.

Advances in next-generation sequencing technology, whole-genome mapping, and bioinformatics improve access to complete genomes, which allow comparative analyses at both the nucleotide and the structural levels. In this study, we compared 31 complete genomes from two recent statewide *B. pertussis* epidemics with vaccine strains and observed the first definitive view of genome structural variation through rearrangement at a nucleotide sequence resolution within this species. These data challenge the previously held view of population clonality, revealing new levels of diversity, even within geographically defined epidemics. As more complete genomic, transcriptomic, and proteomic data are made available for additional strains collected across broader time periods, it will become possible to discern the stability and rate of rearrangements, when certain structural profiles emerged, and whether profiles shift between epidemic and nonepidemic years. Such further analyses are needed to determine how rearrangement events correlate with SNP phylogenetic relationships and aid development of a comprehensive typing methodology that incorporates nucleotide and structural variation to draw meaningful conclusions from associated clinical metadata. It remains unclear if and how rearrangements play a role in adaption and virulence. Genomes of isolates collected from asymptomatic carriers are needed as a baseline, in conjunction with transcriptomic and proteomic analysis, to determine what insertion elements or rearrangements, if any, are specific to increased disease severity in some patients. Although this study cannot link rearrangements to virulence or adaptation, it highlights the need for complete assemblies to fully evaluate the contribution of pathogen evolution, in addition to waning immunity and incomplete protection from current vaccines, toward pertussis reemergence.

## MATERIALS AND METHODS

### Bacterial strains.

Isolates submitted to the California Department of Public Health (CDPH) were received in three manners. First, local health departments passively submitted isolates to CDPH after performing primary isolation or after hospital submission. For *B. pertussis* deaths, CDPH actively reached out to the local health jurisdiction or hospital to determine if an isolate was available and requested submission to CDPH. Additionally, in 2010 local public health laboratories were sent a request for submission to CDPH of any *B. pertussis* isolate that had not been previously submitted. The 13 California isolates sequenced in this study (Table 1) were chosen as part of a collaborative project between CDPH and the CDC focused on determining the phylogenetic relationship of *B. pertussis* isolates from California infants less than 3 months old where *B. pertussis* infection led to either fatal or nonfatal pertussis disease (49). From November 2011 to December 2012, 4,100 nasopharyngeal specimens were received at the Vermont Department of Health Laboratory (VDHL) from pediatric practices and hospitals and tested by culture and, if ordered by the physician, by PCR. During the outbreak, 72% of all PCR-positive specimens were confirmed by culture, of which the VDHL submitted 432 isolates to the CDC in 2013 for molecular characterization. In general, *prn* genotype and PFGE profiles were taken into account to select the most diverse pool of 20 Vermont isolates used for whole-genome sequencing. The CS strain (C393) and Tohama I isolate (E476) were also resequenced in this study (Table 1).

### Genomic DNA preparation.

Isolates were cultured on Regan-Lowe agar without cephalexin for 72 h at 37°C. Genomic DNA isolation and purification were conducted according to the Qiagen Gentra Puregene yeast/bacterial kit standard protocol with slight modification (Qiagen, Valencia, CA). Briefly, two aliquots of approximately 1 × 10^9^ bacterial cells were harvested and resuspended in 500 µl of 0.85% sterile saline and then pelleted by centrifugation for 1 min at 16,000 × *g*. Following the protocol to completion, 100 µl of DNA hydration solution was added to dissolve the genomic DNA. Aliquots were quantified and qualified using a NanoDrop 2000 (Thermo Fisher Scientific Inc., Wilmington, DE) spectrophotometer.

### Genome sequencing and assembly.

Whole-genome shotgun sequencing of each isolate was performed using a combination of the PacBio RSII (Pacific Biosciences, Menlo Park, CA) and Illumina MiSeq (Illumina, San Diego, CA) platforms. Genomic DNA libraries were prepared for PacBio sequencing runs using the SMRTbell template prep kit 1.0 and polymerase binding kit P4, while MiSeq libraries were prepared using the NEB Ultra Library prep kit (New England BioLabs, Ipswich, MA). PacBio sequencing reads were filtered with the following cutoffs: 500-bp minimum subread length, 0.80 minimum polymerase read quality, and 100-bp minimum polymerase read length. *De novo* genome assembly of passed reads was performed first using the Hierarchical Genome Assembly Process (HGAP, v3; Pacific Biosciences) with a 6-kb minimum seed read length, 15× target coverage, 0.06 overlap error rate, and 40-bp minimum overlap length (57). These initial assemblies were further improved by unambiguously mapped PacBio reads with a minimum length of 50 bp and quality score of 75 using BLASR (v1) with a maximum divergence of 30% and minimum anchor of 12 bp (28). The resulting consensus sequences were determined with Quiver (v1), manually checked for circularity, and then reordered to match the start of the CS reference sequence (NC_017223).

Assemblies were confirmed by comparison to restriction digest optical maps (as described below) and further “polished” by mapping Illumina MiSeq PE-150 reads using CLC Genomics Workbench (v7.5; CLC bio, Boston, MA). Raw reads were first trimmed at both ends to remove bases with quality scores less than 0.01 and ambiguous nucleotides and then filtered to remove reads less than 45 bp. The resulting trimmed reads were mapped against the HGAP assembly with the following parameters: mismatch cost of 2, insertion/deletion cost of 3, length fraction of 0.95, similarity fraction of 0.95, and local alignment end gap calculation. Potential errors were identified using the Basic Variant Detection module with the following parameters: maximum coverage of 1,000×, minimum coverage of 10×, minimum read count of 5, minimum variant frequency of 51%, neighborhood radius of 5 bp, minimum central quality score of 25, and minimum average neighborhood quality score of 20. Detect errors were then corrected either manually or with a custom Perl script. Assembly statistics are detailed in Data Set S1 in the supplemental material. Final assemblies were annotated using the NCBI automated Prokaryotic Genome Annotation Pipeline (PGAP).

### Optical mapping.

Optical maps for each isolate were prepared from cells of single 1-mm colony equivalents following growth on Regan-Lowe agar without cephalexin using the Argus system (OpGen, Gaithersburg, MD) according to special company protocols. Briefly, high-molecular-mass bacterial DNA (205-kbp average size) was isolated with minimal shearing and applied to a chemically modified glass surface with fabricated microfluidic channels. The stretched DNA on the channels was digested *in situ* with KpnI in a partial digestion mode and stained with a JoeJoe fluorescent dye on an automatic MapCard processor. To confirm revealed unusual insertions and duplications, restriction enzyme BamHI was used. The digested DNA molecules were imaged using an Argus fluorescence microscope and Path-Finder automated image-acquisition and tiling optical map assembly software (OpGen). The resulting single-molecule restriction maps were assembled into consensus whole-genome maps with Gentig software (OpGen) that recurrently aligned overlapping DNA molecules with similar fragments to calculate a concluding map. Final whole-genome maps in this study are composites from at least 32 single fragmented molecules at every point and typically represent an average depth of 50 to 300 molecules. Restriction map alignments between different strains were generated using MapSolver software (v.2.1.1; OpGen, Gaithersburg, MD). Pairwise alignments were performed between all maps using an alignment score of 3. Optical maps for each of the 33 epidemic isolate and vaccine strain single-contig assemblies were compared to the *in silico* restriction map of the vaccine isolate Tohama I (NC_002929.2) using MapSolver as an independent validation. Optical maps were also compared to *in silico* digests of HGAP assemblies to confirm genome structure, and when necessary, *de novo* assemblies were repeated.

### Whole-genome alignment.

Whole-genome assemblies of epidemic isolates were aligned using progressiveMauve (58) along with C393, E476, and four publicly available complete genomes: CS (NC_017223), Tohama I (NC_002929), B1917 (CP009751), and B1920 (CP009752). Genomes were clustered based on changes in order and orientation of homologous sequence blocks using the MLGO pipeline (59). Multicontig assemblies for I488 and I517 were excluded from whole-genome alignment and rearrangement analyses.

### Virulence gene and variant analysis.

Genome assemblies for all 33 epidemic isolates were compared to E476 (Tohama I) using CLC Genomics Workbench. Coding regions of 44 known virulence genes were found using BLASTn and aligned against E476 using MEGA6 (60) to detect SNPs and predict resulting amino acid changes. All SNPs were confirmed by manual inspection of Illumina read alignments. Additionally, we have proposed the nomenclature of two genes, *fimW* (*fim2*) and *fimH* (*fim3*), to better conform to bacterial nomenclature standards and avoid confusion in allele typing.

Whole-genome variant analysis was performed by mapping from Illumina reads of each isolate to the E476 genome. The mapping parameters were the same as described for genome “polishing” above with a few exceptions. These exceptions include a length fraction of 0.90 and a similarity fraction of 0.90. Variants were determined using the Basic Variant Detection tool in CLC Genomics Workbench. A phylogenetic tree was constructed from single nucleotide variants (SNVs; *n* = 758) and multiple nucleotide variants (MNVs) (*n* = 123) after removal of variants in proximity to IS*481* and IS*1002* insertions. The resulting 408-bp concatenated alignment was used to calculate a maximum likelihood phylogenetic reconstruction with ascertainment bias correction using RAxML v8 (61). Proteins containing nonsynonymous mutations were predicted based on the E476 genome annotation information and then were further classified into functional categories using HMMER v3.1b2 (http://hmmer.org/) (62) to search a betaproteobacterium-specific subset of EggNOG ver. 4.1 (63).

### Nucleotide sequence accession numbers.

The whole-genome shotgun sequences have been deposited at DDBJ/EMBL/GenBank under the accession numbers CP010249 to CP010266, CP010347, CP010838 to CP010847, CP010961 to CP010964, JWLA00000000, and JWLB00000000. The versions described in this paper are the first versions. The project description and related metadata are accessible through BioProject number PRJNA266616.
