# Supplementary figures and images for: Genome Structural Diversity among 31 Bordetella pertussis Isolates from Two Recent U.S. Whooping Cough Statewide Epidemics
Source: mSphere. 2016 May 11;1(3):e00036-16. doi: 10.1128/mSphere.00036-16 (PMC4888882; doi:10.1128/mSphere.00036-16)

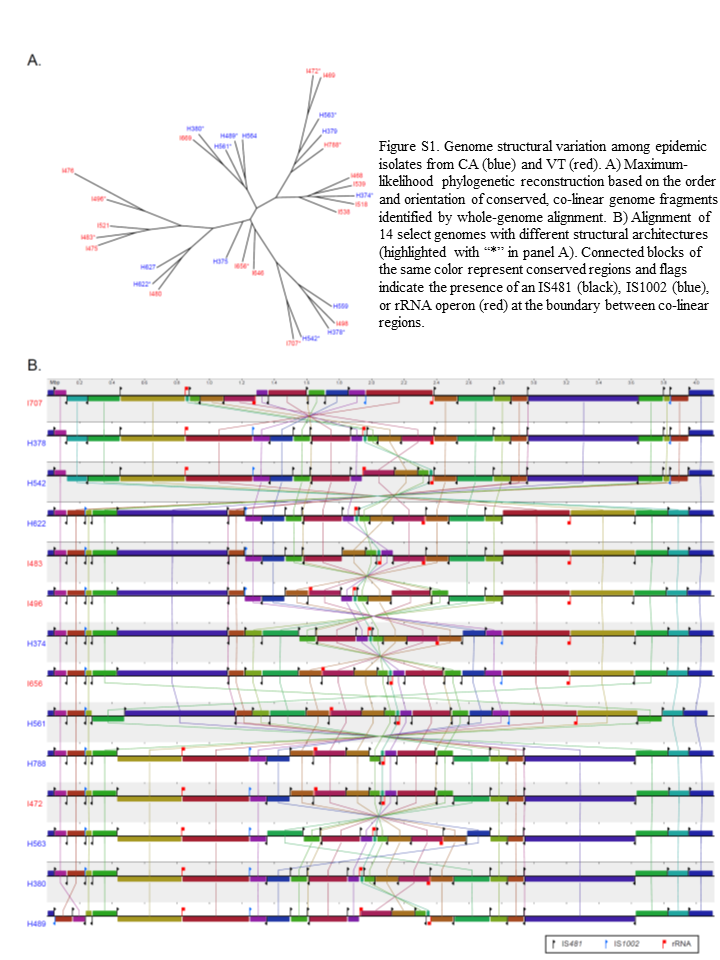

Supplement: Figure S1 [file sph003162078sf1.tif]
